# Effectiveness of early detection on breast cancer mortality reduction in Catalonia (Spain)

**DOI:** 10.1186/1471-2407-9-326

**Published:** 2009-09-15

**Authors:** Montserrat Rue, Ester Vilaprinyo, Sandra Lee, Montserrat Martinez-Alonso, Misericordia Carles, Rafael Marcos-Gragera, Roger Pla, Josep-Alfons Espinas

**Affiliations:** 1 Biomedical Research Institut of Lleida (IRBLLEIDA), Lleida, Catalonia, Spain; 2 University of Lleida, Lleida, Catalonia, Spain; 3 Fundation Dr. Ferran, Hospital of Tortosa, Catalonia, Spain; 4 Dana Farber Cancer Institute, Boston, USA; 5 Rovira i Virgili University, Reus, Catalonia, Spain; 6 Girona Cancer Registry, Girona, Catalonia, Spain; 7 Catalan Cancer Plan. Department of Health, Catalonia, Spain; 8 Catalan Institute of Health, Terres de l'Ebre Region, Catalonia, Spain

## Abstract

**Background:**

At present, it is complicated to use screening trials to determine the optimal age intervals and periodicities of breast cancer early detection. Mathematical models are an alternative that has been widely used. The aim of this study was to estimate the effect of different breast cancer early detection strategies in Catalonia (Spain), in terms of breast cancer mortality reduction (MR) and years of life gained (YLG), using the stochastic models developed by Lee and Zelen (LZ).

**Methods:**

We used the LZ model to estimate the cumulative probability of death for a cohort exposed to different screening strategies after *T *years of follow-up. We also obtained the cumulative probability of death for a cohort with no screening. These probabilities were used to estimate the possible breast cancer MR and YLG by age, period and cohort of birth. The inputs of the model were: incidence of, mortality from and survival after breast cancer, mortality from other causes, distribution of breast cancer stages at diagnosis and sensitivity of mammography. The outputs were relative breast cancer MR and YLG.

**Results:**

Relative breast cancer MR varied from 20% for biennial exams in the 50 to 69 age interval to 30% for annual exams in the 40 to 74 age interval. When strategies differ in periodicity but not in the age interval of exams, biennial screening achieved almost 80% of the annual screening MR. In contrast to MR, the effect on YLG of extending screening from 69 to 74 years of age was smaller than the effect of extending the screening from 50 to 45 or 40 years.

**Conclusion:**

In this study we have obtained a measure of the effect of breast cancer screening in terms of mortality and years of life gained. The Lee and Zelen mathematical models have been very useful for assessing the impact of different modalities of early detection on MR and YLG in Catalonia (Spain).

## Background

Randomized controlled trials (RCT) are the gold standard for measuring medical interventions. Although controversial, RCT assessing the effectiveness of screening with mammography have provided valuable information [1,2]. While there is still debate about the best screening strategies, and what benefits they produce, at present the cost, time, contamination issues and difficulties with compliance preclude additional RCT for early detection of breast cancer. Because statistical population trends are affected by many factors and therefore are not accurate in measuring the effect of health interventions, there has been increased interest in using population data and mathematical models to assess the effectiveness of early breast cancer detection.

Mathematical models for assessing the effect of health interventions are structured representations of health states and the transitions between them. These models may describe the relationship between an intervention and changes in incidence and mortality rates for a specific disease in a particular population. In the United States (US), a consortium of researchers participating in the Cancer Intervention and Surveillance Modeling Network (CISNET) used statistical and simulation modeling to quantify the relative impact of adjuvant therapy and screening mammography on the decline of breast cancer mortality [3,4]. The stochastic models that Sandra Lee and Marvin Zelen designed in the US under CISNET are an alternative to population trials for addressing and responding to most of the questions that arise when developing and assessing the effect of early detection [5-10].

In Spain there is a National Health System (NHS), financed primarily by taxes, which provides universal and free health coverage, including early detection of breast cancer. Catalonia is an autonomous region of Spain which has approximately one sixth of the Spanish population. By the year 2007, the Catalan Health Service was providing services to seven million inhabitants, including 3.5 million women. The Catalan Breast Cancer Screening Program (BCSP) started gradually, at the beginning of the 1990s, providing biennial mammography screening tests, with the target population being women 50-64 years old. Since the year 2000, women older than 64 are kept in the program until the age of 69, based on the results of a model-derived cost-effectiveness study published in 1998 [11]. At the present time, there is interest in assessing the impact and cost-effectiveness of different modalities (age at the first exam, number of exams and periodicity of exams) of breast cancer early detection in Catalonia (Spain).

The aim of this study was to estimate the effect of different strategies of breast cancer early detection in Catalonia (Spain), in terms of reduction of breast cancer mortality and years of life gained, using the stochastic models developed by Lee and Zelen.

## Methods

### The Lee and Zelen's (LZ) model

**Figure 1:**
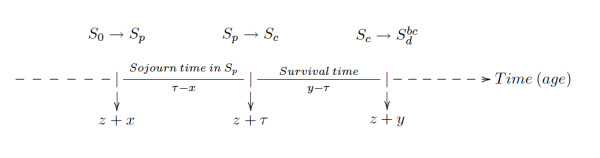
Health states and transitions in the Lee and Zelen's model.

Lee and Zelen developed a probabilistic model that predicts mortality as a function of the early detection modality. The characteristics and assumptions of the LZ model are described in detail elsewhere [5-10]. The assumptions of the LZ model (see Figure 1) are (1) a four-state progressive disease in which a subject may be in a disease-free state (*S*_0_), preclinical disease state (*S*_*p*_: capable of being diagnosed by a special exam), clinical state (*S*_*c*_: diagnosis by typical health care), and a death from breast cancer state ; (2) age-dependent transitions into the different states; (3) age-dependent examination sensitivity; (4) age-dependent sojourn times in each state; and (5) exam-diagnosed cases have a stage-shift in the direction of more favorable prognosis relative to the distribution of stages with typical health care.

The LZ model considers:

• *n *screening exams at times *t*_0 _<*t*_1 _< ... <*t*_*n*-1_. It is assumed that *t*_0 _= 0 and age = *z *at *t*_0 _= 0.

• Three chronological times (see Figure 1):

- *x*: time at entering *S*_*p*_, *z *+ *x*: age when entering *S*_*p*_. The time *x *is not observed but can be derived from the incidence function and the distribution of sojourn time in the *S*_*p *_state. *x *takes a negative value if the transition to *S*_*p *_occurs before the age at first exam, *z*.

- *τ*: time at entering *S*_*c*_, *z *+ *τ *: age at entering *S*_*c*_. The time *τ *can not be observed in cases detected by exam, only in the clinically detected cases. For cases detected by exam, *τ *can be estimated.

- *y*: time at death: *x *<*τ *<*y*

• Sojourn time in *S*_*p*_: *τ *- *x*

• Sojourn time in *S*_*c*_: *y *- *τ*

A very relevant element that the LZ models use is the concept of an individual of generation *j*, which is defined as an individual that enters the pre-clinical state at the *j*th interval, (*t*_*j*-1_, *t*_*j*_). The formulas used to estimate the model probabilities are based on this concept.

The LZ basic model calculates the cumulative probability of death for the cohort group exposed to any screening program after *T *years of follow-up. Similarly, the cumulative probability of death for the cohort group having typical health care can be calculated. These probabilities are used to calculate the possible reduction in mortality from an early detection program after *T *years of follow-up.

Survival distributions for exam-diagnosed, interval, and control cases are assumed to be conditional on the stage at diagnosis and treatment, but are not dependent on the mode of diagnosis. The LZ model assumes *k *stages, *ϕ*_*s*_(*j*), *ϕ*_*i*_(*j*) and *ϕ*_*c*_(*j*) represent the probability of being diagnosed at stage *j*, *j *= 1,..., *k *for exam-diagnosed, interval and control cases, respectively, and *f*_*j*_(*t*|*z *+ *τ*) is the probability density function (pdf) of survival time *t *among subjects who would have been clinically diagnosed at stage *j *in the absence of screening. Then the survival time *pdf*s of the exam-diagnosed, interval and control cases are the mixtures ,  and , respectively.

Since screening will appear to increase survival time, the LZ model controls for *lead time *bias by setting the origin of survival time for the screened, interval, and clinical cases at the time of clinical diagnosis. Consequently, there is an implied *guarantee time *for disease-specific survival, that is, the cases diagnosed earlier would have been alive at the time the disease would have been clinically diagnosed. This guarantee time, also called *lead time*, is a random variable and is incorporated into the equations of the model.

Explicitly, the lead time is *τ *- *t*_*r *_where *τ *is the time at which the individual enters the clinical state and *t*_*r *_is the time at which the *r *detection exam, when the disease will be diagnosed, is given.

### The inputs of the Lee and Zelen's model for Catalonia

We studied women born during the calendar years 1930 to 1959. In this article we present results for the cohorts born from 1955 to 1959. We assumed that the incidence of breast cancer for ages younger than 25 years was insignificant. When incidence or mortality data were not available, they were estimated using age-cohort models [12].

#### Incidence of breast cancer in Catalonia

Incidence data from the population-based cancer registries of the Catalan provinces Girona and Tarragona was used. These two registries cover 20% of the Catalan population. Data from the province of Girona was provided by the Girona Cancer Registry and data from Tarragona was downloaded from the International Agency for Research on Cancer (IARC) [13]. The available periods with information on breast cancer incidence were 1980-1989 and 1994-2002 for Girona and 1983-1997 for Tarragona. We obtained the observed incidence rate by combining both sources of information and using the population counts of the official census for the same time periods [14]. To model the data and obtain incidence estimates outside of the observed calendar years we used a generalized linear model with a Poisson distribution and polynomial parametrization of age and cohort variables [15]. This model included a forth-degree polynomial for the age effects and a second-degree polynomial for the cohort effects. Models with lower degree terms for the age or cohort effects did not fit the observed data properly.

#### Mortality due to causes other than breast cancer in Catalonia

We used the multi-decrement life table methodology to partition overall mortality into mortality due to breast cancer and mortality due to causes other than breast cancer [16]. Mortality data was obtained from the Catalan Mortality Registry and the National Institute of Statistics (INE) [14,17]. Overall mortality data was available for calendar years 1900 to 2004 and breast cancer mortality data was available for calendar years 1975-2004. Population estimates were obtained from the INE and the Catalan Statistics Institute (IDESCAT) [14,18]. We subtracted the conditional probabilities of dying from breast cancer from the overall conditional probabilities of death to obtain the probabilities of dying from causes other than breast cancer. We estimated the missing breast cancer mortality probabilities for earlier years of birth using an age-cohort model similar to the generalized linear model described in the incidence data section. Details of these estimations can be found elsewhere [19].

#### Distribution of stages at diagnosis

Since there was limited information in Catalonia on the distribution of disease stages at diagnosis, we used the US data. Lee and Zelen [7] reported the AJCC distributions of stages for cases diagnosed without screening, screening-detected cases and interval cases. The stage distribution for cases without screening was provided by the Surveillance Epidemiology and End Results (SEER) program of the National Cancer Institute and the stage distribution for the screen-detected and interval cases were provided by the Breast Cancer Surveillance Consortium (BCSC). For screen-detected cases and interval cases Lee and Zelen distinguish between annual, biennial and irregular screening. Details on stage distribution and definitions of screen-detected or interval cases can be found in Lee and Zelen's work.

#### Sensitivity of mammography

The sensitivity of mammography in our model was assumed to be the following: 0.55 for < 40 years, 0.65 for 40 - 45 years, 0.70 for 45 - 50 years, 0.75 for 50 - 70 years and 0.80 for ≥ 70 years. These values were used by Lee and Zelen for screening exams conducted in 1995-2000, when they estimated the impact of mammography and adjuvant treatments in the US [7]. Lee and Zelen derived these data from the BCSC database which contains mammogram screening data and follow-up for approximately one million US women starting from 1994.

#### Estimation of survival functions in Catalonia

Breast cancer survival data was not available, in Catalonia, at the population level. We obtained breast cancer survival data from the Girona province Cancer Registry, which covers an area of 700,000 inhabitants and represents approximately 10% of the Catalan population. Since data from the Girona Registry was scarce, to obtain stable estimates of the Catalan survival functions by age group and disease extension, we derived the Catalan survival time (*pdf*s) from survival data for the US in calendar years 1975-1979. First, based on the Girona Cancer Registry and the US data, we estimated the hazard ratios for Girona in the period 1980-1989 versus the US in the period 1975-79, by AJCC disease stage. Both periods are considered prior to the dissemination of screening mammography in the respective countries and survival functions in these periods are not affected by the lead time bias. Second, we multiplied the estimated hazard ratios by the US hazard rates, by age and stage of disease, and obtained an estimate of the Catalan hazard functions for the 1980-1989 period. We assumed that survival data from the Girona Cancer Registry is representative of Catalan breast cancer survival. Fourth, we used the Catalan hazard functions to obtain cumulative survival functions and survival time *pdf*s.

If the hazard ratios of Girona versus the US were not proportional over time, we estimated a time dependent hazard ratio using the formula:(1)

The Catalan cumulative survival functions, by stage of disease, obtained from the estimated hazard functions using expression (1) fit the observed data from the Girona Cancer Registry well, based on the deviance statistic.

More details on how we obtained the Catalan breast cancer survival functions can be found elsewhere [20].

We used the same method to obtain estimates of the survival functions for the 1990-2001 period and we used these functions to asses the impact of changing the survival functions in the effectiveness of early detection. Since screening was prevalent in Catalonia during the 1990s, these survival functions are affected by the lead time bias and overestimate survival time after breast cancer diagnosis. In this paper we used them as a very favourable scenario to compare with results obtained when using the survival functions of the 1980s.

### The application of Lee and Zelen's model to assess the effect of different breast cancer screening scenarios in Catalonia

#### Estimation of mortality for the not-screened group (control group)

Lee and Zelen [5] estimated *I*_*c*_(*y*|*z*), the probability of dying *y *years after the start of the study conditional on being age *z *at time zero for the group receiving usual care as:

where *i*(*z *+ *τ*) is the age-specific point incidence function for age *z *+ *τ *and *g*_*c*_(*y *- *τ*|*z *+ *τ*) is the survival time *pdf *of control detected cases.

In the LZ model, the probability of disease-specific death, for the control group, at age *z *+ *T *can be estimated as:

And, the *cumulative probability of disease-specific death*, for the control group, after *T *years of follow-up time can be estimated as:

#### Estimation of mortality for the screened group

##### Mortality from cases detected in the screening exams

Lee and Zelen estimate the probability *D*_*r*_(*y*|*z*) of being diagnosed at the *r *exam (time *t*_*r*_) and dying *y *years after the start of the study, where *z *is the age at the start of the study. In order to obtain *D*_*r*_(*y*|*z*) Lee and Zelen distinguish two situations:

1. Being diagnosed at the first exam (*t*_0_, *r *= 0). In this case the women had entered *S*_*p *_before *t*_0_.

2. Being diagnosed at subsequent exams *r *= 1,... *n *- 1. In this case there are three possible situations depending on cases:

(a) being at *S*_*p *_before *t*_0_. All the previous screening exams gave false negative results.

(b) entering *S*_*p *_at a later time *x *after *t*_0_, but prior to the exam *r *- 1 (time *t*_*r*-1_), (*t*_*j*-1 _<*x *≤ *t*_*j*_, *j *= 1, 2,..., *r *- 1, *r *> 1). At least the exam *r *- 1 gave a false negative result.

(c) entering *S*_*p *_at (*t*_*r*-1_, *t*_*r*_). No previous false negative results.

The four situations cover all the possibilities of early detection in a specific exam. Adding up the cumulative probabilities of dying in any of these four situations, one obtains the probability that a diagnosed case dies after *y *years of having started the study:

##### Mortality from cases detected in intervals between exams

Similarly to cases detected by exams, one can estimate the probability *I*_*r*_(*y*|*z*) of being diagnosed in the *r *interval between exams (*t*_*r*-1_, *t*_*r*_) and dying *y *years after the start of the study.

Once the probabilities *I*_*r*_(*y*|*z*) are estimated, the probability that an interval case dies *y *years after the start of the study is:

Combining both possibilities of detection, the probability of disease-specific death for cases diagnosed in the early detection program, at age *z *+ *T*, can be estimated as:

And the *cumulative probability of disease-specific death *for cases diagnosed in the early detection program, after *T *years of follow-up time, is:

### Measures of effect

#### Relative breast cancer mortality reduction (MR) up to a specific age

For a specific cohort *ν*, the breast cancer relative mortality reduction up to age *w*, can be obtained using the expression:

In our analysis we have considered the upper limit of age *w *to be 80 years. MR has been obtained from 40 to 80 years of age for the cohort.

#### Years of life gained (YLG) up to a specific age

To obtain the average number of years of life gained (*Y LG*) attributable to screening, we subtracted the number of years of life lost due to breast cancer with screening (*Y LL*) from years of life lost without screening (*YLL*_0_). The number of years lost have been computed as zero for the proportion of women that survive up to 80 years of age, and as the difference between 80 and the age at death (*z *+ *y*) for the proportion of women that die from breast cancer. Women that do not die from breast cancer are assumed not to have lost any years of life. Thus, the *YLG *up to age 80, have been estimated as:

with *I*_*c*_(*y*|*z*), *D*(*y*|*z*) and *I*(*y*|*z*) defined as in the MR section.

Then, *YLG *per woman screened have been estimated by dividing the number of *YLG *by the proportion of women surviving at age 40 in the Catalan population. Similarly, *YLG *per breast cancer diagnosed have been estimated by dividing *YLG *by the proportion of women with a diagnosis of breast cancer in the interval 40 to 80 years of age.

### Sensitivity analysis

In order to assess the impact on mortality reduction of changes in the input parameters, we varied the mammography sensitivity and the survival time *pdf*s. We estimated the impact of changing the sensitivity of mammography by setting the initial sensitivities in the model to 90% for all age groups. But, since changes in the sensitivity of mammography may affect the distribution of stages at diagnosis as well as the distribution of sojourn time in a pre-clinical state, for which we do not have accurate data, we present only data on the effect of changing the survival time *pdf*s.

## Results

### Effect of early screening on mortality reduction (MR)

Table 1 presents the effect of early detection on breast cancer mortality MR (first column), in the 40 to 80 year age interval, for cohorts born in the period 1955-59, with the following assumptions: 1) screening started in 1985, 2) all women in the target population participated in early detection and 3) survival *pdf*s are the estimated Catalan *pdf*s for the 1980-1989 period. Measures of effect were obtained by comparing the screened cohort to a cohort with the same characteristics, but without screening. In Table 1 we show the data obtained with screening for the age intervals 40-74, 40-69, 50-74 and 50-69, both with annual and biennial mammography exams.

**Table 1:** Estimated effect of early breast cancer (BC) detection, in the 40-80 year age interval, with different screening strategies.

Periodicity	Age interval for screening exams	Effect of early detection		
		MR (%)	YLG per woman screened	YLG per BC detected
Annual	40-74	29.8	0.222	1.874
Biennial	40-74	22.9	0.165	1.387
				
Annual	45-74	28.9	0.205	1.727
Biennial	45-74	22.3	0.152	1.283
				
Annual	50-74	27.4	0.180	1.515
Biennial	50-74	21.3	0.134	1.127
				
Annual	40-69	27.6	0.219	1.845
Biennial	40-69	21.2	0.162	1.369
				
Annual	45-69	26.7	0.201	1.697
Biennial	45-69	21.2	0.151	1.272
				
Annual	50-69	25.3	0.176	1.485
Biennial	50-69	19.6	0.132	1.109

Relative BC mortality reduction (MR) and Years of Life Gained (YLG). Catalonia, cohorts born in 1955-59.

For annual screening in the 40-74, 45-74 and 50-74 age intervals, the estimated MR were 29.8%, 28.9% and 27.4%, respectively. When annual screening was done in the 40-69, 45-69 and 50-69 age intervals, the estimated MR were 27.6%, 26.7% and 25.3%, respectively. When strategies differ in periodicity but not in the age interval of exams, biennial screening achieves almost 80% of the annual screening MR. For example, annual exams in the age interval 40-74 for women born in 1955-59 represent a MR of 30%, whereas biennial exams in this age group represent a 23% MR.

### Effect of early screening on years of life gained (YLG)

Table 1 (second and third columns) presents the average number of YLG per woman participating in early detection and per breast cancer detected, respectively, in the 40-80 year age interval. Gains per woman screened range from 0.13 years (48 days) for biennial exams in the 50-69 age interval to 0.22 years (80 days) for annual exams in the 40-74 age interval. Gains per breast cancer detected extend from 1.11 years for biennial exams in the 50-69 age interval to 1.87 years for annual exams in the 40-74 age interval. In contrast to MR, the effect on YLG of extending screening from 69 to 74 years of age is much smaller than the effect of extending the screening from 50 to 45 or 40 years.

### Impact of changing the survival probability density functions (pdfs) on the estimated effect of early detection

Table 2 shows the effect on MR and YLG when different survival *pdf*s are used. Results for annual exams in ages 40-74 years and biennial exams in ages 50-69 years are presented. In scenario A we assumed that the survival time *pdf*s were the 1980-1989 Catalan functions (pre-mammography period). In scenario B we assumed an improvement in survival was due to the utilization of adjuvant treatments (hazard ratio of 0.75 with reference to the 1980-89 Catalan hazard rates). In scenario C we used the Catalan survival *pdf*s for 1990-2001, which correspond to the post-mammography-introduction period and overestimate survival because of the inclusion of the lead time.

**Table 2:** Estimated effect of early detection of breast cancer (BC), in the 40-80 year age interval, with different BC survival scenarios.

	Scenario A		Scenario B		Scenario C	
Interval of exams	Annual	Biennial	Annual	Biennial	Annual	Biennial
	MR (%)					

40-74	29.8	22.9	32.5	25.1	44.0	33.8
50-69	25.3	19.6	27.6	21.3	36.7	27.6

	YLG per woman screened					

40-74	0.222	0.165	0.203	0.151	0.208	0.154
50-69	0.176	0.132	0.161	0.120	0.162	0.119

	YLG per breast cancer detected					

40-74	1.874	1.387	1.711	1.272	1.753	1.300
50-69	1.485	1.109	1.353	1.010	1.367	1.003

Relative BC mortality reduction (MR) and years of life gained (YLG). Catalonia, cohorts born in 1955-59.

A: Survival probability density functions (*pdf*s): Catalonia 1980-1989 (pre-dissemination of mammography screening).

B: Resulting survival *pdf*s after applying a hazard ratio of 0.75 to the 1980-1989 hazard rates, in order to take into account the effect of adjuvant treatments.

C: Survival *pdf*s: Catalonia 1990-2001 (survival functions have not been corrected by the lead time bias that originates from earlier detection). This scenario produces an overestimation of the effect of early detection.

Table 2 shows an improvement in mortality reduction for both screening strategies when the survival functions improve. If the hazard rates for women diagnosed with breast cancer decrease to 75% of the 1980-89 hazard rates (scenario B), MR would increase about 2 percentage points. When using the 1990-2001 survival functions (scenario C), MR increases dramatically, reaching values of 44% for annual screening in the 40-74 age interval and 27.6% for biennial screening in the 50-69 age interval. These values overestimate MR because of the lead time bias, but show that the effect of early detection is influenced by the survival distribution used in the model. The higher the differences in survival between stage I and stages II- and II+, the higher the reduction in mortality attributable to screening.

When looking at the YLG between 40 and 80 years of age, we did not see the same pattern as with MR (Table 2). Contrary to what was expected, YLG per woman screened or per breast cancer diagnosed remained similar or even decreased when the survival functions improved. We attribute this result to the fact that, when survival by stage of disease improves, there is a gain in life-years in the no-screening group, as well.

## Discussion

Randomized clinical trials are important for assessing the effects of screening. Nevertheless, the benefit of screening for breast cancer has remained controversial because of inconsistent results from clinical trials and controversies in systematic reviews [1,2]. Mathematical models may help answer questions for which empirical evidence is scarce and aid in understanding some of the basic issues relating to the early diagnosis of breast cancer [5]. We have identified mathematical models which are very useful for assessing the impact of different modalities of early detection on reducing mortality and potential years of life lost in Catalonia (Spain). Among the different approaches to population modeling, we chose the Lee and Zelen model because its assumptions are realistic and consistent with other data sources [5,10]. The LZ model is flexible, can incorporate complex information and interventions and may be used to determine optimal screening modalities.

Our aim was to assess the effect of different early detection scenarios using data from Catalonia, when available. We used Catalan population and mortality statistics. Breast cancer incidence was estimated using data from Cancer Registries in two Catalan provinces (Girona and Tarragona) and survival after a diagnosis of breast cancer was obtained using data from one of the Catalan Cancer Registries (the Girona Cancer Registry) and the US survival data. Incidence and mortality data for future time periods was projected using age-cohort models. When regional data was not available, we used information from the literature. We assumed that screening started in 1985, which is consistent with the fact that the Catalan Breast Cancer Screening Program (BCSP) started gradually at the beginning of the 1990s, but some opportunistic breast cancer screening was done in the public and private health care sector during the 1980s [21]. We also assumed that exams started at 40 years of age or later and ceased at 69 or 74 years of age. In order to compare the effect of different modalities of screening, we assessed the effect of different screening scenarios in the same age span, 40-80 years of age. Our results reflect the effect of early detection if all women in each specific cohort had participated and complied with the screening scenarios assessed.

## Our findings

For all the studied birth cohorts, depending on the screening scenario, our estimated reduction in breast cancer mortality in the 40-80 age span varied from about 20% for biennial exams in the 50-69 age interval to about 30% for annual exams in the 40-74 age interval. When exams were performed biennially, the MR achieved was almost 80% of the annual screening MR.

With annual exams, extending the program from 69 to 74 years produced mortality reductions roughly 2% higher, whereas extending the program from 50 to 45 years produced increases of 1.5%. Extending from 45 to 40 years represented an increase of 1% in mortality reduction. If we look at the years of life gained, there were mimimal changes when the program was extended from 69 to 74 years of age, whereas extending the program from 50 to 40 years of age increased the time gained per breast cancer diagnosed by about 0.3 years (four months). In any case, these results should be interpreted with caution, because the impact of early detection also needs to take into account the potential harm and cost of intensive screening [22-24]. We have also observed that changes in breast cancer survival have an impact on the mortality reduction achieved but not on the years of life gained.

Changes in the sensitivity of mammography to 90% sensitivity in all age groups did not result in changes in MR or YLG (data not shown). As we mentioned in the methods section, an improvement in mammography sensitivity may affect the distribution of the stages at diagnosis and the distribution of sojourn time in a pre-clinical state. Since we did not change these distributions, our assessment of the impact of changing the sensitivity of mammography may not fully reflect what would happen in practice.

## Comparison with other studies

Tabar *et al*, using data from the Regional Oncology Centres and Statistics Sweden for two Swedish counties (Dalarna and Linkping), compared all deaths from breast cancer diagnosed in the 20 years before screening was introduced with those in the 20 years after introduction of screening [25]. After adjustment for age, self-selection bias, and changes in breast-cancer incidence in the 40-69 year age-group, Tabar *et al *estimated a 44% reduction in breast cancer mortality in women exposed to screening (RR = 0.56). They estimated a 16% reduction in women not exposed to screening (RR = 0.84). The 33.3% ((0.84-0.56)/0.84) difference in breast cancer mortality reduction between the screened and non-screened group can be interpreted as the reduction attributable to screening. This figure is higher than our estimated 21% reduction in the 40-69 year age-group with biennial exams, a scenario comparable to the study of two Swedish counties where the screening interval was 18 months for women in the 40-54 year age-group and two years in older women [26]. Similarly, the 39% reduction reported by the Swedish Organised Service Screening Evaluation group, using data from six counties with interscreening intervals of ≃ two years, is higher than our result for annual screening in the 40-69 year age interval [27,28].

Anderson *et al *assessed the impact of early detection in Connecticut. They found that incidence rates for early-stage tumors increased dramatically, whereas rates for late-stage tumors experienced a modest decrease. Breast cancer mortality rates fell 31.6%. These results were consistent with effective early detection and improved treatment over time, but also suggest that many mammography-detected early-stage lesions may never progress to late-stage cancers [29].

A Cochrane systematic review updated in 2006 by Gotzsche and Nielsen, based on seven trials involving half a million women in Europe and North America, estimated a 15%-20% reduction in breast cancer mortality [24]. These trials were conducted in the 1970s and the 1980s, and had different age intervals for exams and different periodicities. Most of the trials included women aged 50 to 69 and the periodicity was variable; in most of them screening was nearer to biennial exams than annual. Reductions obtained using the LZ models with the Catalan 1980-89 data are consistent with the results reported in the Cochrane review.

## Limitations and other considerations

We may have overstated the advantages of modelling and the inability of RCT to answer some specific questions, such as age at initiation and the ideal frequency of screening. It should be noted that many of the parameters used in the models are obtained from the published RCT. In fact, the models could also be seen as a form of sensitivity analysis around health policy decision making. Models can complement RCT by testing hypotheses that would be impossible to test otherwise.

The assumption that the distribution of breast cancer stages at diagnosis was the same in Catalonia as in the US may have slightly biased our results. We made this assumption because information on the distribution of stages for different screening patterns was not available in Catalonia. We know that, before the introduction of mammography, the distribution of stages in the GCR was worse than in the US (41% localized, 49% regional and 10% distant stages in the GCR in the 1980-89 period versus 53% localized, 38% regional and 9% distant stages in the US in the 1975-79 period). If we assume that the stage distribution for different screening scenarios in Catalonia is similar to the US distribution (which is consistent with most of the RCT results, 70-80% of early diagnosed cases which are node negative), then the stage shift attributable to screening would have been even higher in Catalonia, resulting in a larger effect of early detection. Therefore, the assumption we made about the distribution of breast cancer stages at diagnosis may have resulted in a underestimation of the effects.

We do not present a validation of our findings in this article comparing the estimated effect of screening with observed mortality data. Breast cancer mortality in the last two decades has not only been influenced by the introduction of mammography but also by other events like the dissemination of adjuvant treatments. To validate our findings we need to incorporate the dissemination of mammography and adjuvant treatments, similar to what the CISNET did in the US [3]. We are planning to do that soon.

Several authors have reported a significant increase in incidence attributable to screening [29,30]. We used an age-period-cohort model to assess changes in incidence trend related to the dissemination of screening. We found a slight increase in incidence starting at the beginning of the 1990s that did not reach statistical significance. As a consequence, we projected breast cancer incidence using an age-cohort model.

Gotzsche and Nielsen estimated a 30% increase in overdiagnosis and overtreatment [24]. According to these authors, for every 2000 women invited for screening throughout 10 years, one will have her life prolonged and 10 healthy women, who would not have been diagnosed if there had not been screening, will be diagnosed as breast cancer patients and will be treated unnecessarily.

This study has not estimated the impact of false positive results of early detection exams. We think that this is an important issue to take into account when assessing the effect of early detection. We are planning to extend the LZ models to estimate the impact of false positive screening results. Some authors have estimated that after 10 years of annual screening, 30 to 50% of women have at least one false-positive mammogram result [31-33]. In Catalonia, Castells *et al *estimated that after 10 mammograms, the cumulative false positive recall rate was 32.4% [34].

Finally, the ultimate goal of our project is to assess the cost-effectiveness of different strategies for the early detection of breast cancer in Catalonia. In this study we have obtained a measure of the effect of breast cancer screening in terms of mortality and years of life gained. The impact of false positives and a cost-effectiveness analysis using an extension of the LZ models are our next targets.

## Conclusion

We have estimated the impact of different strategies for early detection, using mammography, on breast cancer mortality reduction. For the first time our study presents a measure of the effect of early detection based on the observed Catalan incidence and breast cancer survival data. Since it is currently difficult to use experimental studies to determine optimal age intervals and periodicities for screening, mathematical models are an alternative for assessing the effects of early detection.

## Competing interests

The authors declare that they have no competing interests.

## Authors' contributions

MR: Co-developed the project that includes this study, performed statistical analysis, wrote drafts and obtained author feedback.

EV: Developed the computer programs that estimate the effect of screening under different scenarios. Provided statistical analysis and interpretation of results. Participated in the writing and revising of the manuscript.

SL: Provided guidance and advise during the development of the study. Participated in the writing and revising of the manuscript.

MMA: Developed the age-cohort models to estimate incidence and mortality. Participated in the statistical analysis of results and interpretation. Participated in the writing of the manuscript.

MC, RP and JAE: Co-developed the project that includes this study, participated in writing and revising the manuscript.

RMG: Provided the survival data, participated in the statistical analysis and in writing and revising the manuscript.

All authors read and approved the final version of the manuscript.

## Pre-publication history

The pre-publication history for this paper can be accessed here:

http://www.biomedcentral.com/1471-2407/9/326/prepub

## References

[B1] Olsen O Gotzsche PC Screening for breast cancer with mammography Cochrane Database Syst Rev 2001 4 CD001877 11687128 10.1002/14651858.CD001877

[B2] de Koning HJ Mammographic screening: evidence from randomised controlled trials Ann Oncol 2003 14 1185 1189 10.1093/annonc/mdg319 12881373

[B3] Berry DA Cronin KA Plevritis SK Fryback DG Clarke L Zelen M Mandelblatt JS Yakovlev AY Habbema JD Feuer EJ Cancer Intervention and Surveillance Modeling Network (CISNET) Collaborators Effect of screening and adjuvant therapy on mortality from breast cancer N Engl J Med 2005 353 1784 1792 10.1056/NEJMoa050518 16251534

[B4] Feuer E Plevritis SK Berry DA Cronin KA eds Cancer Intervention and Surveillance Modeling Network (CISNET) Collaborators The impact of mammography and adjuvant therapy on US breast cancer mortality (1975-2000): collective results from the Cancer Intervention and Surveillance modeling network J Natl Cancer Inst Monogr 2006 36 1 126

[B5] Lee SJ Zelen M Mortality Modeling of Early Detection Programs Biometrics 2008 64 386 395 10.1111/j.1541-0420.2007.00893.x 17725809

[B6] Zelen M Lee SJ Models and the early detection of disease: methodological considerations Cancer Treat Res 2002 113 1 18 12613347 10.1007/978-1-4757-3571-0_1

[B7] Lee S Zelen M A stochastic model for predicting the mortality of breast cancer J Natl Cancer Inst Monogr 2006 36 79 86 17032897 10.1093/jncimonographs/lgj011

[B8] Lee SJ Zelen M Modelling the early detection of breast cancer Ann Oncol 2003 14 1199 1202 10.1093/annonc/mdg323 12881377

[B9] Lee S Huang H Zelen M Early detection of disease and scheduling of screening examinations Stat Methods Med Res 2004 13 443 456 10.1191/0962280204sm377ra 15587433

[B10] Lee S Zelen M Scheduling periodic examinations for the early detection of disease: applications to breast cancer J Am Stat Assoc 1998 93 1271 1281 10.2307/2670042

[B11] Beemsterboer PMM Warmerdam PG Boer R Borras JM Moreno V Viladiu P de Konning HJ Screening for breast cancer in Catalonia. Which policy is to be preferred? Eur J Public Health 1998 8 241 246 10.1093/eurpub/8.3.241

[B12] Holford TR The estimation of age, period and cohort effects for vital rates Biometrics 1983 39 311 324 10.2307/2531004 6626659

[B13] International Agency for Research on Cancer CANCER Mondial 2007 http://www-dep.iarc.fr

[B14] Instituto Nacional de Estadística Population data 2007 http://www.ine.es

[B15] McCullagh P Nelder JA Generalized linear models 1983 Monographs on statistics and applied probability, London-New York: Chapman and Hall

[B16] Rosenberg M Competing risks to breast cancer mortality J Natl Cancer Inst Monogr 2006 36 15 9 17032889 10.1093/jncimonographs/lgj004

[B17] Generalitat de Catalunya Departament de Sanitat i Seguretat Social Direcció General de Recursos Sanitaris Anàlisi de la mortalitat a Catalunya 1983-2006 http://www.gencat.net/salut/

[B18] Institut d'Estadística de Catalunya Padró municipal d'habitants 2007 http://www.idescat.net

[B19] Vilaprinyo E Gispert R Martinez-Alonso M Carles M Pla R Espinas JA Rue M Competing Risks to Breast Cancer Mortality in Catalonia BMC Cancer 2008 8 331 doi:10.1186/1471-2407-8-331. 10.1186/1471-2407-8-331 19014473 PMC2636833

[B20] Vilaprinyo E Rue M Marcos-Gragera R Martinez-Alonso M Estimation of age- and stage-specific Catalan breast cancer survival functions using US and Catalan survival data BMC Cancer 2009 9 98 doi:10.1186/1471-2407-9-98. 10.1186/1471-2407-9-98 19331670 PMC2679763

[B21] Rue M Carles M Vilaprinyo E Martinez-Alonso M Espinas JA Pla R Brugulat P Dissemination of periodic mammography and patterns of use, by birth cohort, in Catalonia (Spain) BMC Cancer 2008 8 336 doi:10.1186/1471-2407-8-336. 10.1186/1471-2407-8-336 19014679 PMC2613154

[B22] Stout NK Rosenberg MA Trentham-Dietz A Smith MA Robinson SM Fryback DG Retrospective cost-effectiveness analysis of screening mammography J Natl Cancer Inst 2006 98 774 782 16757702 10.1093/jnci/djj210

[B23] Woo PP Kim JJ Leung GM What is the most cost-effective population-based cancer screening program for Chinese women? J Clin Oncol 2007 25 617 624 10.1200/JCO.2006.06.0210 17308266

[B24] Gotzsche PC Nielsen M Screening for breast cancer with mammography Cochrane Database Syst Rev 2006 4 CD001877 17054145 10.1002/14651858.CD001877.pub2

[B25] Tabar L Yen MF Vitak B Chen HH Smith RA Duffy SW Mammography service screening and mortality in breast cancer patients: 20-year follow-up before and after introduction of screening Lancet 2003 361 1405 1410 10.1016/S0140-6736(03)13143-1 12727392

[B26] Tabar L Fagerberg CJ Gad A Baldetorp L Holmberg LH Grontoft O Ljungquist U Lundstrom B Manson JC Eklund G Reduction in mortality from breast cancer after mass screening with mammography. Randomised trial from the Breast Cancer Screening Working Group of the Swedish National Board of Health and Welfare Lancet 1985 1 829 832 10.1016/S0140-6736(85)92204-4 2858707

[B27] Swedish Organised Service Screening Evaluation Group Reduction in breast cancer mortality from organized service screening with mammography: 1. Further confirmation with extended data Cancer Epidemiol Biomarkers Prev 2006 15 45 51 10.1158/1055-9965.EPI-05-0349 16434585

[B28] Swedish Organised Service Screening Evaluation Group Reduction in breast cancer mortality from the organised service screening with mammography: 2. Validation with alternative analytic methods Cancer Epidemiol Biomarkers Prev 2006 15 52 56 10.1158/1055-9965.EPI-05-0953 16434586

[B29] Anderson WF Jatoi I Devesa SS Assessing the impact of screening mammography: Breast cancer incidence and mortality rates in Connecticut (1943-2002) Breast Cancer Res Treat 2006 99 333 340 10.1007/s10549-006-9214-z 16703451

[B30] Holford TR Cronin KA Mariotto AB Feuer EJ Changing patterns in breast cancer incidence trends J Natl Cancer Inst Monogr 2006 36 19 25 17032890 10.1093/jncimonographs/lgj016

[B31] Blanchard K Colbert JA Kopans DB Moore R Halpern EF Hughes KS Smith BL Tanabe KK Michaelson JS Long-term risk of false-positive screening results and subsequent biopsy as a function of mammography use Radiology 2006 240 335 342 10.1148/radiol.2402050107 16864665

[B32] Christiansen CL Wang F Barton MB Kreuter W Elmore JG Gelfand AE Fletcher SW Predicting the cumulative risk of false-positive mammograms J Natl Cancer Inst 2000 92 1657 1666 10.1093/jnci/92.20.1657 11036111

[B33] Elmore JG Barton MB Moceri VM Polk S Arena PJ Fletcher SW Ten-year risk of false positive screening mammograms and clinical breast examinations N Engl J Med 1998 338 1089 1096 10.1056/NEJM199804163381601 9545356

[B34] Castells X Molins E Macia F Cumulative false positive recall rate and association with participant related factors in a population based breast cancer screening programme J Epidemiol Community Health 2006 60 316 321 10.1136/jech.2005.042119 16537348 PMC2593411

